# Observed and Modeled Seasonal Air Quality and Respiratory Health in Senegal During 2015 and 2016

**DOI:** 10.1029/2019GH000214

**Published:** 2019-12-06

**Authors:** Nafissatou Oumar Toure, Ndeye Ramatoulaye Diagne Gueye, Aminata Mbow‐Diokhane, Gregory S. Jenkins, Maggie Li, Mamadou S. Drame, Karen Adjoa Ronke Coker, Khady Thiam

**Affiliations:** ^1^ Université Cheikh Anta Diop Faculté de Médecine de Pharmacie et d'Odontologie Dakar Senegal; ^2^ UFR des Sciences de la Santé de l'Université de Thies, Thies Senegal; ^3^ Centre de Gestion de la Qualité de l'Air, Direction de l'Environnement et des Etablissements Classés Dakar Senegal; ^4^ Department of Meteorology and Atmospheric Science, Pennsylvania State University University Park PA USA; ^5^ Currently at Department of Environmental Health Sciences Columbia University Mailman School of Public Health New York NY USA; ^6^ Faculté des Sciences et Techniques Université Cheikh Anta Diop Dakar Senegal; ^7^ School of International Affairs, Pennsylvania State University University Park PA USA; ^8^ Currently at Department of Environmental and Global Health University of Florida College of Public Health and Health Professions Gainesville FL USA

**Keywords:** Particulate matter, dust, asthma, bronchitis, ARI, Senegal

## Abstract

In this work, we use existing particulate matter (PM) data from Dakar, Senegal, satellite aerosol optical depth (AOD) and the Weather Research and Forecasting (WRF) model to evaluate the role of dust transport from the Sahara and PM concentrations and exposure into other administrative districts of Senegal during 2015 and 2016. We also use data from the Ministry of Health to examine spatial and temporal patterns of acute respiratory infections, asthma, bronchitis, and tuberculosis across Senegal with an emphasis on Northern Hemisphere winter December–February, when air quality is poor, and June–August when there is an improvement in air quality. Measurements in Dakar, Senegal, suggest hazardous PM_10_ concentrations associated with Saharan dust storms but lower PM_10_ concentrations during the summer. The WRF dust simulations show a similar temporal pattern to the observations in Dakar, Senegal, with notable biases. However, the WRF model suggests that the highest dust concentrations are found across the northern half of Senegal during the winter season where there are no currently PM measurements. Health data during 2015–2016 show the highest prevalence of asthma and bronchitis in Dakar, Senegal, suggesting that other sources of air pollution are important. Acute respiratory infection is prevalent throughout the country with the high prevalence found in rural zones, for children between 12 and 59 months. All measures including real‐time monitoring, air quality forecast, and communication should be used to protect the public from potentially hazardous environmental conditions during the winter season.

## Key Points

- Saharan dust transport is responsible for poor air quality over Senegal from attribution dust simulations of the WRF model
- During 2015 and 2016, asthma and bronchitis prevalence are highest in the administrative district of Dakar during the summer season (JJA)
- ARI prevalence exceeds 9,000 per 100,000 for children between 12 and 59 months in the administrative districts of Kafferine during DJF of 2015–2016

## 1 Introduction

Senegal is located in the Sahel with a latitude range of 12 and 16.5°N and a longitude range of 17.5 and 12°W. It has a semiarid climate with a wet season (June–October) and a dry season (November through April). Senegal has numerous ethnic groups with an estimated 14.3 million inhabitants, respectively, in 2016. The Gross Domestic Product of Senegal is 14.8 billion USD for 2015. The United Nations Human Development Index, which includes factors such as education, poverty, health, and equity, ranked Senegal 170 out of 187 countries in 2015. Senegal has developed strategies to address many issues such as food security, water resources, public health, and poverty alleviation but is currently denoted as a low‐income country. The World Health Organization defines One Health as the linkage amongst the environment, human, and animal health. Changing environmental conditions from weather to climate scales have affected human health in West Africa and over much of the African continent including Senegal.

Environmental studies across the Sahel (Senegal in the western Sahel) over the last five decades have focused on summer season rainfall variability, which was associated with a multidecadal drought during the 1970s and 1980s (Nicholson, 1993), which has shown some recovery. The causes of the drying period have been linked to sea surface temperature anomalies and potentially anthropogenic climate change (Giannini et al., 2013) and dust emissions from the Sahara Desert (Prospero & Lamb, 2003). The recovery of Sahelian rainfall over the last few decades have been associated with less frequent rain events but more intense rainfall events along with warmer daytime and nighttime temperatures (Mouhamed et al., 2013). There have been far fewer studies on long‐term trends or anomalies in winter season dust concentrations, but there is evidence that dust concentrations were higher during the 1980s based on visibility, satellite, proxy data, and modeling studies (Evan et al., 2016; Jenkins & Gueye, 2018; Mbourou et al., 1997; Silue et al., 2013). Dust modeling studies using the WRF model simulate more transport from the Sahara leading higher dust concentrations across the Sahel during 1988–2003, but lower dust concentrations between 2003 and 2014 (Jenkins & Gueye, 2018).

Air pollution, in the form of particulate matter (PM), can be natural or anthropogenic (transportation, industrial, biomass burning, indoor cooking, and fires) occurring within households and the ambient environment negatively impacting human health. Indirectly, air pollution is responsible for approximately one and nine deaths globally, and ARIs are a leading cause of mortality amongst children less than 5 years old (Landrigan et al., 2018; Roy, 2016; World Health Organization (WHO), 2016). Heft‐Neal et al. (2018) suggest that PM is strongly linked in infant mortality in Africa, with PM_2.5_ linked to a considerable fraction of infant mortality in West Africa. A number of studies in Senegal have linked air pollution to respiratory disease (Sylla, Faye, Fall, Lo, et al., 2017; Sylla et al., 2018). Sylla, Faye, Fall, & Anta, 2017provide a review of studies in Africa linking air pollution to cardiovascular and respiratory disease.

Saharan dust is a nonanthropogenic source of PM of varying size ranges and is a source of poor air quality, with large quantities of dust transported into the Sahel region of West Africa exposing millions of people to hazardous PM concentrations. The health effects of Saharan dust exposure on human health are not well quantified in West Africa (de Longueville et al., 2013). Observed Saharan dust events in Senegal, produce hazardous PM_10_ and PM_2.5_ concentrations, which can promote respiratory and cardiovascular disease (Diokhane et al., 2016; Marticorena et al., 2010). Zhang et al. (2016) summarize health effects of dust globally and find that desert dust is associated with respiratory disease (asthma and pneumonia) and cardiovascular disease (stroke, ischemic heart disease, and cerebrovascular disease). However, there is also evidence that there are only minor causal linkages between asthma and Sahara dust in Barbados (Prospero et al., 2008) and that reported asthma cases are more likely in the wet season in Nigeria (Aderele, 1979).

During the dry season, when Saharan dust is present there is evidence that dust exposure can drive an infectious disease such as measles and meningitis (Agier et al., 2013; Bharti et al., 2012; Martigny & Chiapello, 2013) across the Sahel. For example, in 2012, Diokhane et al. (2016) found a higher number of suspected meningitis cases in Senegal when compared to 2013. Higher meningitis cases occurred when measured dust concentrations were higher on average during the winter months in 2012 compared to 2013, and hazardous dust concentrations are found with dust events from late February through mid‐March. Respiratory pathogens can exist on the surface of dust particles and can also serve to increase respiratory disease (Griffin, 2007; Kellogg et al., 2004).

Increased indoor activity because of poor air quality during the winter season or heavy rains during the summer season in West Africa may also indirectly contribute to the spread of tuberculosis (TB), which is rapidly increasing across Africa. In 2015, the World Health Organization African region (47 of 53 countries in Africa) had an estimated 2,720,000 new TB cases. Men, women, and children under 14 years of age accounted for 54.4%, 35.2%, and 10.36% of the cases nearly mirroring the global values. Consequently, many countries have national TB programs to limit and eventually eliminate the disease. Other factors responsible for TB include urbanization, population density, income, gender, age, contact with infected persons, smoking versus nonsmoking, diabetes, indoor ventilation, migration, HIV positive status, and indoor pollution (Lönnroth et al., 2009).

Senegal is exposed to hazardous PM_10_ and PM_2.5_ dust concentrations during the winter and spring seasons, with events that can last for several days (Diokhane et al., 2016; Gueye & Jenkins, 2019; Marticorena et al., 2010). During the summer season, the dust is lifted above the monsoon layer and typically found between 1 and 5 km based on Lidar observations (Léon et al., 2009). Consequently, there may be direct or indirect linkages between the environment and respiratory health, between winter and summer seasons. A significant limitation across the Sahel is the lack of surface PM observations. For example, in 2012, Dakar, Senegal, was the only location across the Sahel undertaking daily PM measurements, and consequently, the spatial and temporal variability of surface PM_10_ is poorly quantified at national, regional, and continental scales for Africa (WHO, 2016). To address this issue, we use simulated dust concentrations to estimate dust concentrations across Senegal's 14 administrative districts.

We assert that elevated PM_10_ and PM_2.5_ concentrations during the NH winter season could directly or indirectly increase noncommunicable respiratory diseases such as asthma and bronchitis along with ARIs in Senegal. Hence, the objectives of this work are to (a) show the observed seasonal estimates of PM in Senegal for the period of 2013–2016; (b) compare observed PM_10_ and PM_2.5_ concentrations to modeled PM_10_ and PM_2.5_ dust concentration in Senegal for the same period; (c) spatially distribute modeled PM_10_ and PM_2.5_ dust concentration across the 14 administrative districts of Senegal during the winter and summer seasons during 2015–2016 to determine the average dust concentration in each administrative districts during 2015–2016; (d) determine the number of days during the winter and summer seasons that unhealthy dust concentrations are present in the 14 districts during 2016; and (e) determine the spatial and temporal (seasonal) patterns of asthma, bronchitis, ARI, and TB in the 14 administrative districts of Senegal.

## 2 Data Description and Methodology

### 2.1 Atmospheric Data and WRF Simulations of Dust

To examine the environmental dust amounts, we use PM_10_ measurements from Dakar, Senegal (Diokhane et al., 2016), ground‐based aerosol optical depth (AOD) Sun photometer measurements from Mbour, Senegal, and area averaged 1° × 1°degree satellite Moderate Resolution Imaging Spectroradiometer TERRA Deep Blue estimates of AOD over Senegal (Ginoux et al., 2010; Platnick et al., 2015). AOD measurements from Aerosol Robotic Network site (Holben et al., 1998) are located at Mbour, Senegal (14°N, 16°W). PM_10_ and PM_2.5_ concentrations for 2013–2016 are taken at Dakar, Senegal (14.59°N, 17.5°W) in four locations and averaged to produce daily values. PM_10_ and PM_2.5_ measurements are collected by the Centre de Gestion de la Qualité de l'Air, from the Direction de l'Environnement et des Etablissements Classés (Ministry of Environment and Sustainable Development) by Centre de Gestion de la Qualité de l'Air for the period of 2013–2016. However, there are missing PM_2.5_ data during the period and consequently, we do not compute monthly averages if there are less than 20 days of data. We use the WRF model version 3.4 using the GOCART aerosol module to simulate dust PM_10_ and PM_2.5_ concentrations during 2015 and 2016 for a 1° × 1° area focused on Dakar, Senegal, with a configuration similar to previous studies (Jenkins & Diokhane, 2017; Jenkins & Gueye, 2018). The WRF model has a horizontal grid spacing of 18 km and 27 vertical levels and is driven at lateral boundaries by the National Oceanic and Atmospheric Administration National Centers for Environmental Prediction Final analysis for the 2‐year period every 6 h, and aerosols are transported into Senegal from the Sahara Desert during winter months. The direct effect of aerosols is taken into account with reductions in downward solar radiation from aerosol scattering. Dust is removed only by dry deposition in this study, and wet deposition is turned off, which should lead to excessive dust aerosols during the wet season where precipitation acts as a removal process.

### 2.2 Health Data

The health system of Senegal is organized in a pyramidal structure at three levels: central (Ministry of Health), intermediate (Medical Regions), and peripheral called health districts. The Sanitary Data Warehouse of Senegal (EDS‐SN) was designed from the District Health Information System (DHIS2), which is a modular, free, and open system. It was developed as part of a research and development project of the Computer Science Department of Norway's OSLO University called the Health Information System Project. DHIS 2 is geared toward the management of statistical data from peripheral areas to the central level. The health data allow us to analyze respiratory diseases from different regions of Senegal at monthly timescales. In this work, we examine monthly asthma, bronchitis, and ARI data from the 14 administrative districts across Senegal by month for 2015 and 2016. The analysis examines gender, age, seasonal, and spatial characteristics of the three respiratory diseases. We also examine trimester tuberculosis (TB) for adults and children in Senegal from 2013 through 2016. Further, we analyze daily TB cases for the Dakar area from Fann National Hospital, which is located in Dakar, Senegal, during 2016. Geographic Information System (GIS) is used to show the geographic distribution for asthma, bronchitis, and ARI prevalence during the winter and summer season periods.

### 2.3 Demographic and Spatial Data

The national age structure population density data for 2014 were collected from the WorldPop project (Alegana et al., 2015). This mapping approach for this raster data set estimates the number of people in each 5‐year age group per grid square across Senegal, with a spatial resolution of approximately 0.00833333 decimal degrees or 100 m at the equator (Linard et al., 2012). The 5‐year age proportions were developed by Tatem et al. (2013).

To conduct a spatial analysis of environmental, health, and demographic data, we integrated the data with an administrative district boundaries shapefile for Senegal, acquired from the Humanitarian Data Exchange. These data were collected from the Senegalese government and cleaned and *p* coded by the United Nations Office for the Coordination of Humanitarian Affairs Regional Office for West and Central Africa and National Aeronautics and Space Administration Integrated Test and Operations System.

### 2.4 Computing Dust Exposure (Days/Season where Daily Averages Are Over 35 μg/m^3^ for PM_2.5_ and 155 μg/m^3^ for PM_10_)

The 18‐km‐resolution WRF model used in this project estimates different indices of air quality (temperature, humidity, and PM) twice per day. Raster files representing time slices are extracted from the 18‐km‐resolution netCDF WRF model data sets using the Export each time slice from a NetCDF layer as a single raster (*.tif) tool. Each available day of the study from all months in 2015 and 2016 contains two instantaneous times at 0000 and 1200 UTC of PM each day. Average PM_10_ and PM_2.5_ are calculated for each time slice by running a zonal statistics table function with boundaries defined by administrative district lines, with output tables stored as database files (.dbf). These database files are converted into geodatabase file types (.gdb) to enable further editing. Each pair of twice‐daily time slices is averaged to estimate daily PM_2.5_ and PM_10_ for each administrative district. These daily estimates are appended into tables for PM_10_ and PM_2.5_ per season, with the PM_10_ and PM_2.5_ estimates for every day of the winter December–February (DJF) months and summer (June–August, JJA) months.

A copy of each seasonal table is created. A conditional statement is written to convert the daily PM estimated values into binaries contingent on whether each daily value lied above (1) or below (0) the threshold concentrations of 155 μg/m^3^ for PM_10_ and 35 μg/m^3^ for PM_2.5_, as set by the United States Environmental Protection Agency indicating unhealthy air quality for persons that are sensitive. These binaries were summed up in a new column, which was then divided by the total number of days in that 3‐month season to calculate seasonal exposure, quantified by the percentage of days with unhealthy concentrations of PM_2.5_ and PM_10_.

### 2.5 Computing Seasonal ARI, Asthma, and Bronchitis Prevalence (Reported Cases/100,000 Individuals)

The total reported ARI, asthma, and bronchitis outcomes for the winter months of DJF and summer months of JJA 2015 and 2016 data sets are categorized into three bins: children under age 5, children aged 5–15, and all individuals aged 15–60. The age group population density data from 2014 is summarized on the district boundary. A total age group population statistic is calculated for each 5‐year age group per district, representative of annual population within that 5‐year age group, by implementing the zonal statistics as table tool on age structure density data. The percentage of cases per total district population is then calculated by dividing the total number of cases per district (for DJF and JJA 2015 and 2016) by the 2014 age group district population data. The prevalence per district per month for the 0–5 age group, 5–15 age group, and 15–60 age groups is calculated by multiplying the percentage by 100,000 to yield the number of cases per 100,000 individuals.

## 3 Results

There are three ways to estimate the intensity of Saharan dust events in West Africa: (1) surface PM concentrations, (2) satellite estimates of AOD and other optical properties, and (3) surface Sun photometer estimates of AOD and optical properties. Only surface PM concentrations can directly determine the potential impacts of air quality on human health because it represents the ground truth particulate concentrations. However, evaluating air quality from surface measurements in West Africa is challenging because of the lack of continuous measurements and poor spatial coverage. While satellites provide the greatest spatial coverage and surface‐based Sun photometers provide the best temporal sampling, these techniques typically estimate the amount of dust loading in the entire column and not necessarily at the surface. In addition, the dust loading cannot be determined at nighttime or when clouds obscure the Sun. Next, we evaluate air quality over Senegal based on PM measurements in Dakar, Senegal, Moderate Resolution Imaging Spectroradiometer satellite overpasses and Aerosol Robotic Network Sun photometer AOD measurements in Mbour, Senegal.

### 3.1 Annual Observations of PM at Dakar, Senegal

**Figure 1:**
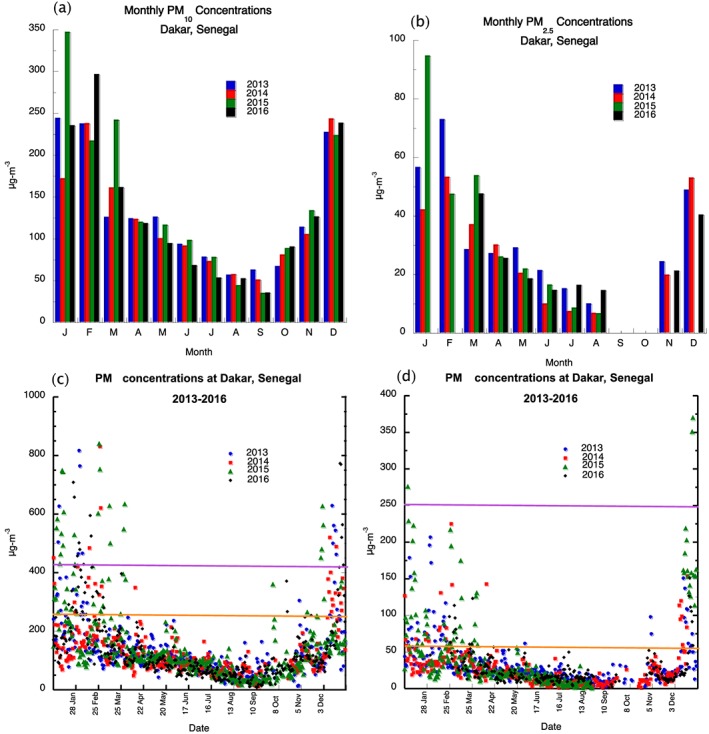
A monthly PM_10_ concentrations, b monthly PM_2.5_ concentrations, c daily PM_10_ concentrations, and d daily monthly PM_2.5_ concentrations for Senegal. PM_2.5_ and PM_10_ units are μg/m^3^.

**Figure 2:**
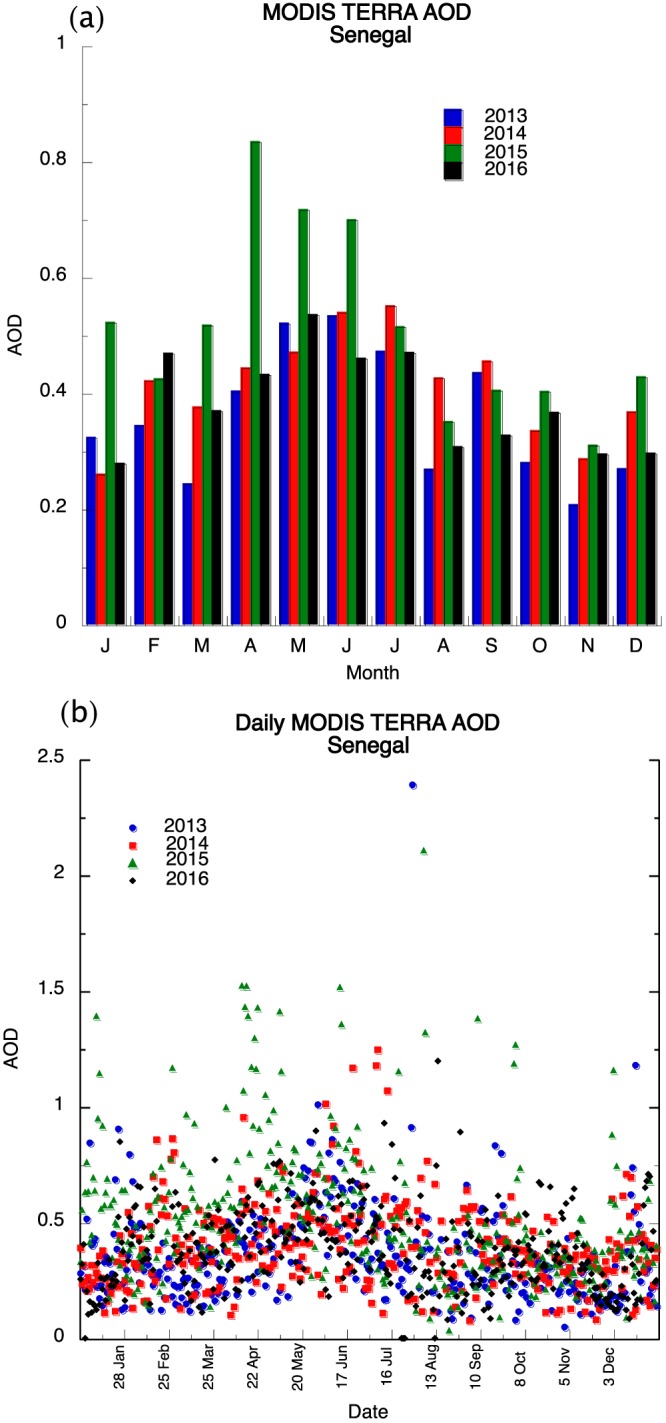
MODIS AOD (2013–2016) for Senegal a monthly and b daily values.

Figures 1a and 1b show the monthly PM_10_ and PM_2.5_ concentrations across Dakar, Senegal for 2013–2016. In general, the highest PM_10_ concentrations are found during the months of December, January, and February with declining values into the summer months. Monthly values during the winter season have a range of 175 to approximately 340 μg/m^3^. Winter season monthly PM_10_ concentrations values are considered unhealthy to sensitive individuals (>155 μg/m^3^) to unhealthy (>255 μg/m^3^), while PM_10_ concentrations fall to moderate levels (< 55 μg/m^3^) during the summer month. Monthly PM_2.5_ concentrations follow a similar trend as PM_10_ with the highest values observed in the winter months. Unhealthy PM_2.5_ concentrations for sensitive groups (> 55 μg/m^3^) are reached during the winter season and decrease to moderate (<35.5 μg/m^3^) and good (<12.5 μg/m^3^) concentrations during the summer month. Daily PM_10_ concentrations frequently exceed unhealthy levels and even hazardous levels from December through March with the highest values exceeding 800 μg/m^3^ during the period (Figure 1c); unhealthy PM concentrations are rarely found during the months of April through September. PM_2.5_ concentrations can exceed unhealthy concentrations for sensitive groups (>55 μg/m^3^), but hazardous concentrations (> 250 μg/m^3^) only occurred 3 times during the four‐year period (Figure 1d). In a similar manner, PM_2.5_ concentrations rarely exceed unhealthy concentrations from April to October. Figure 2a shows AOD monthly measurements for the country of Senegal for 2013–2016 with the highest values typically occurring between April and June. This is opposite to the surface PM measurements but suggests that dust loading is found in the Saharan air layer and located above the monsoon layer (Carlson & Prospero, 1972). The daily AOD values show that while it is possible high AOD values during the winter months, the values are generally less than 1.5 compared to values exceeding 2 during the summer season (Figure 2b).

### 3.2 Comparison Between Observed and Simulated PM During 2015–2016

**Figure 3:**
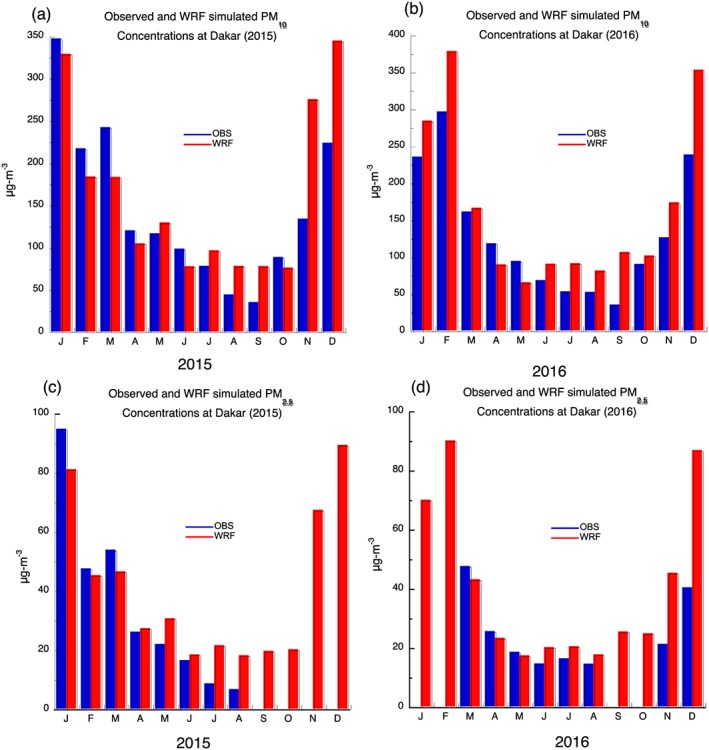
Monthly observed and simulated: A 2015 PM_10_ concentration, b 2016 PM_10_ concentration, c 2015 PM_2.5_ concentration, and 2016 PM_2.5_ concentration. Units are μg/m^3^. Missing blue bars for PM_2.5_ are due to lack of observed data.

Monthly observed and simulated WRF PM_10_ and PM_2.5_ concentrations are compared for Dakar during 2015 and 2016 when respiratory health data are available. The WRF simulated PM_10_ data at Dakar is determined by averaging all grid points with a 1° × 1° box around the city of Dakar. The largest observed PM_10_ concentrations are found during November through March in 2015 and 2016, and this trend is captured in the model although the model tends to underestimate PM_10_ concentrations from January through June 2015 but overestimate monthly values from July 2015 through 2016 (Figure 3a). The temporal patterns are captured in the model simulations with high concentrations during the winter season and reduced PM_10_ concentrations during the summer season. For available station data, monthly PM_2.5_ concentrations are unhealthy during January of 2015 and decrease after April of 2015 to moderate and good levels by July of 2015 (Figure 3c). A similar pattern is found during 2016 for available data, with increasing PM_2.5_ concentration found in November and December of 2016 (Figure 3d). The WRF model shows high dust concentration during January through March time period, with reduced amounts during the summer season followed by a significant increase in PM_2.5_ during November and December of 2015. Simulated values of PM_2.5_ are highly larger during the summer season, most likely because wet deposition is turned off in the WRF simulations. During 2016, high PM_2.5_ dust concentrations are found during January, February, November, and December, although the simulated values are near twice the amount observed at Dakar during December (Figure 3d).

**Figure 4:**
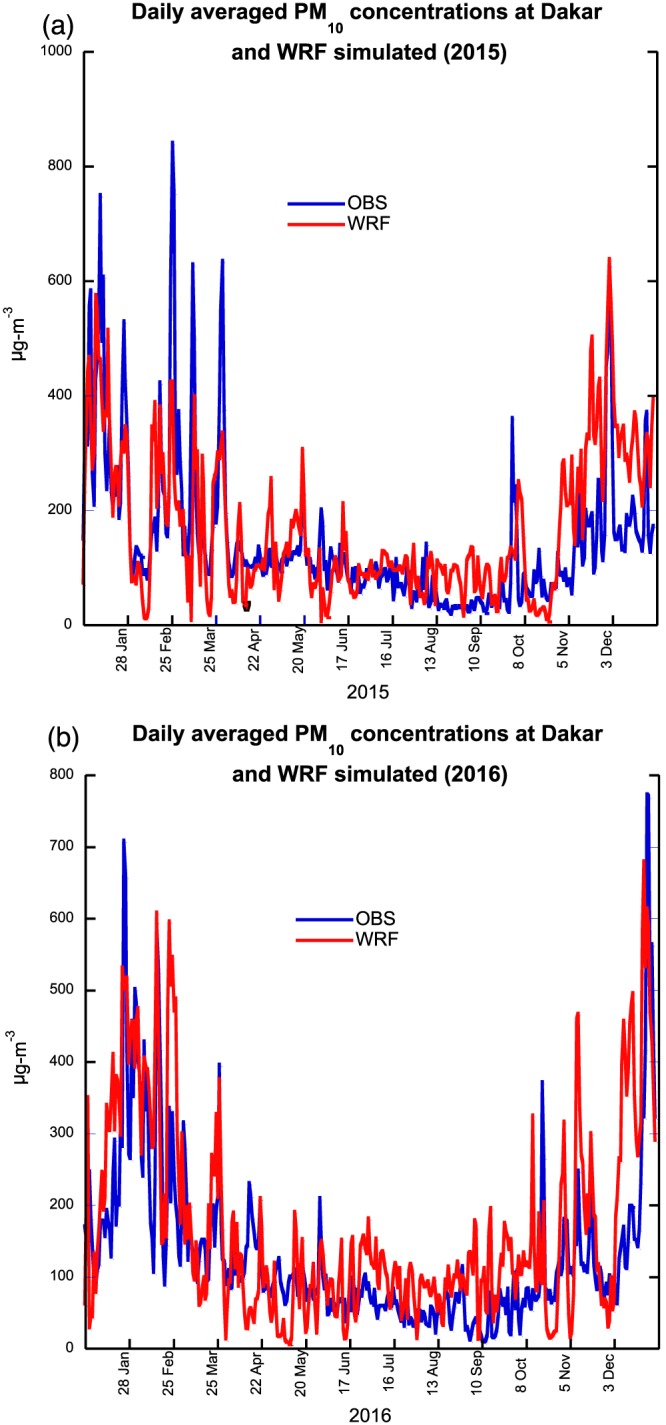
Daily observed and simulated PM_10_ concentrations for a 2015 and b 2016.

Figures 4a and 4b show daily observed and simulated PM_10_ concentrations for 2015 and 2016 for Dakar; we did not use PM_2.5_ because of many missing days during 2016. During the winter season, the observed data show very large dust events where PM_10_ concentrations exceed 800 μg/m^3^ during the 2015 and approach 800 μg/m^3^ during December 2016. The simulated PM_10_ concentrations underestimate very large dust events during January–March 2015 but overestimate dust concentrations during November–December 2015, which is responsible for the large positive bias in monthly values in 2015. In 2016, WRF tends to overestimate the magnitude of the dust events, especially during late February through mid‐March. The second period of overestimated dust concentrations is during early November and then December when simulated PM_10_ concentration exceed observed values by 200–300 μg/m. On an annual basis, the correlations between observed and simulated PM_10_ dust concentrations are 0.70 for 2015 and 0.75 for 2016.

### 3.3 WRF Spatial Distribution of DJF and JJA PM Concentrations and Exposure in Senegal

**Figure 5:**
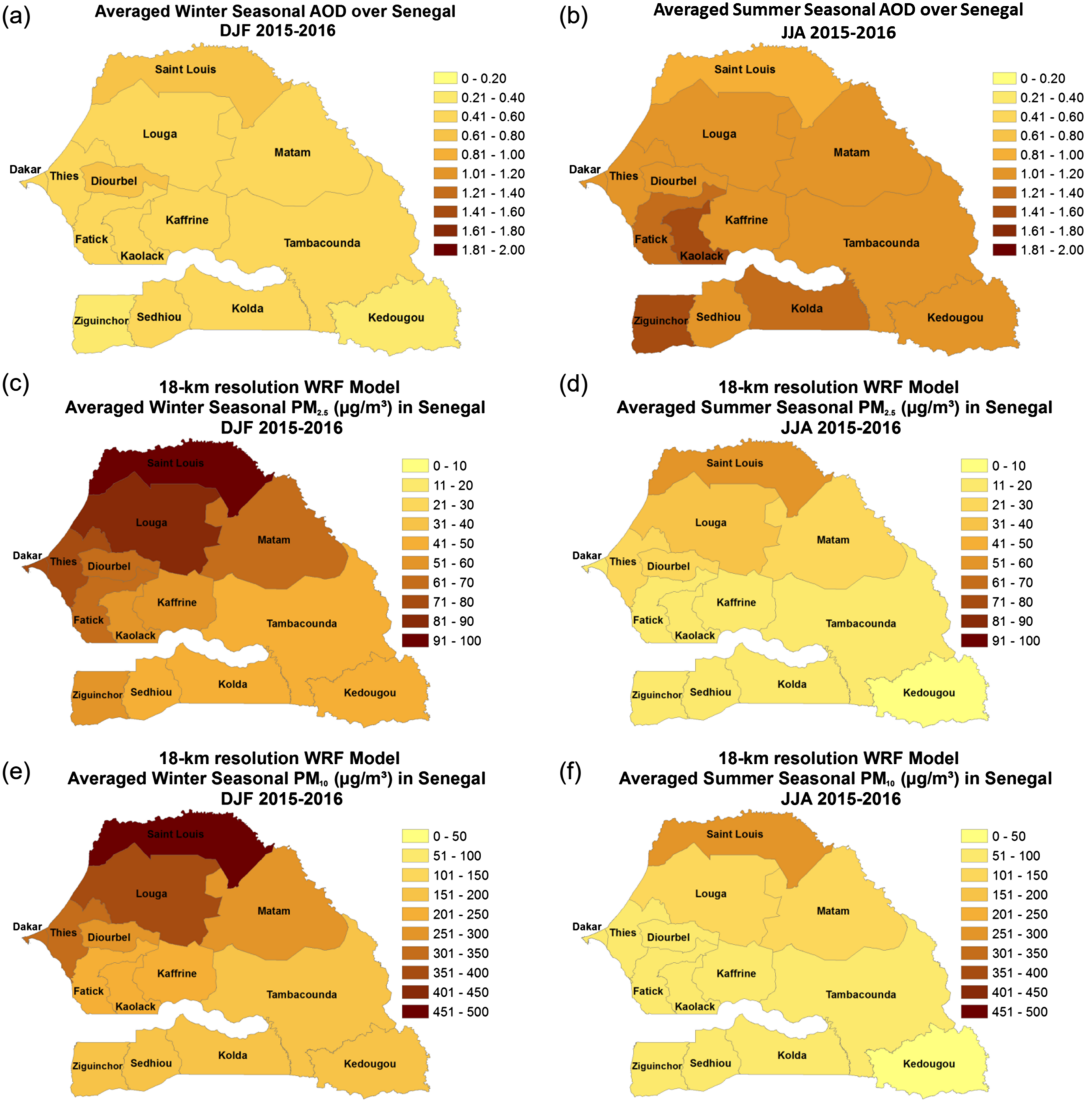
Seasonal simulated spatial distribution of a DJF AOD, b JJA AOD, c DJF PM_2.5_ concentration, d JJA PM_2.5_ concentration, e DJF PM_10_ concentration, and f JJA PM_10_ concentration.

Figures 5a and 5b show the DJF and JJA 2015–2016 averaged AOD distributed throughout the 14 administrative districts of Senegal. The highest AOD values are found in Saint Louis and Diourbel administrative districts during the winter seasons with values greater than 0.6, while the lowest values are found in the southern administrative districts of Ziguinchor and Kedougou. In contrast, during JJA the highest AOD values are found over the central and southern districts of Fatick, Kaolack, Ziguinchor, and Kolda; however, any dust would be found at higher altitudes above the monsoon layer. Simulated PM_2.5_ concentrations during DJF 2015–2016 show the highest PM_2.5_ dust concentrations over the northern administrative districts of Saint Louis and Louga with unhealthy air quality for sensitive groups (>35 μg/m^3^) is found. Simulated PM_2.5_ dust concentrations are significantly reduced during JJA with the lowest values found in the southeastern administrative district of Kedougou; PM_2.5_ remain unhealthy for sensitive groups in the northern administrative district of Saint Louis. PM_10_ dust concentrations exceeding very unhealthy concentration are found over the northern administrative districts of Senegal similar to PM_2.5_ during DJF with improvements in simulated air quality across all of Senegal during JJA (Figures 5e and 5f).

**Figure 6:**
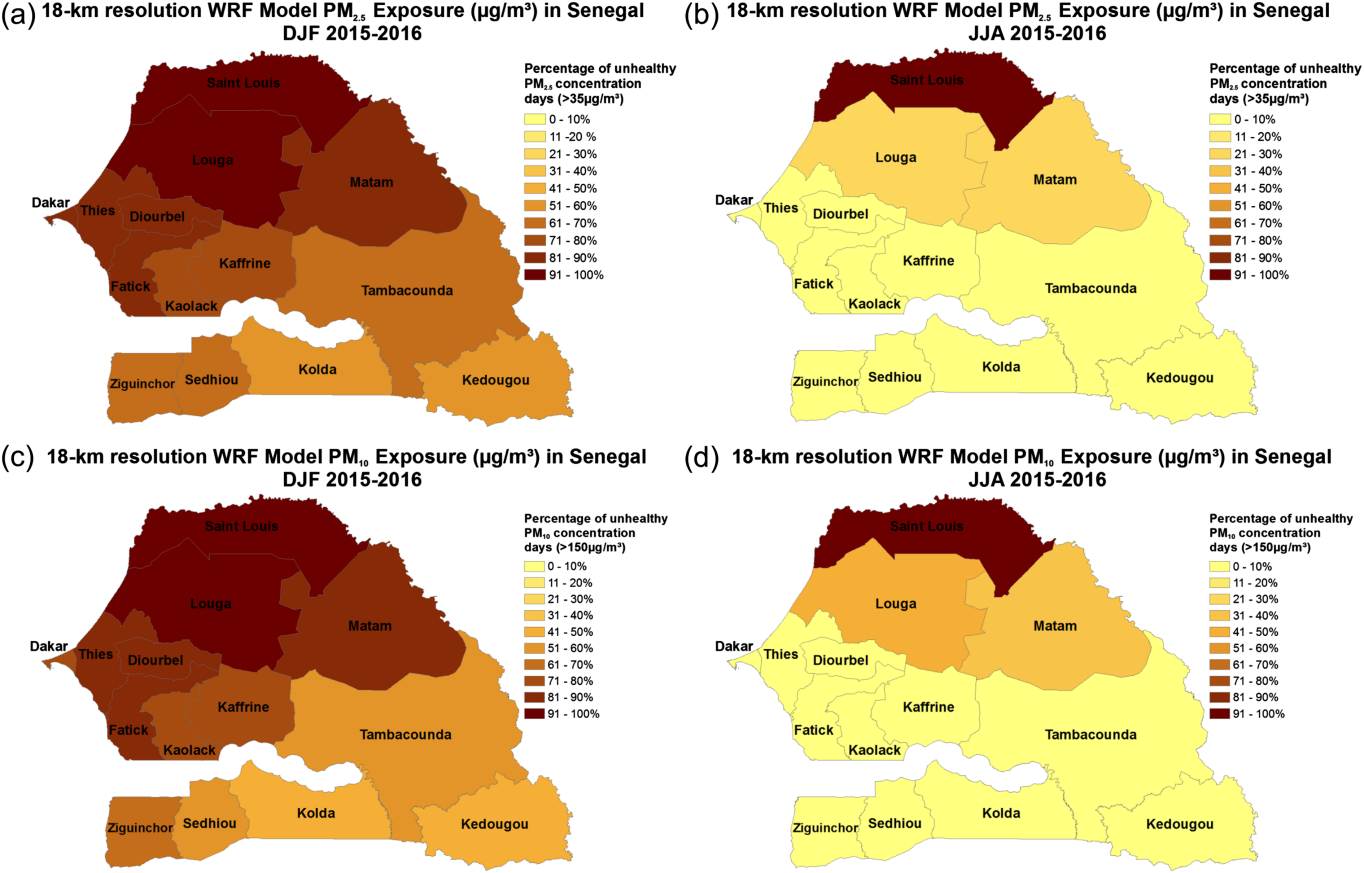
WRF‐simulated unhealthy dust exposure for sensitive groups expressed as percentage of total days in 2015 and 2016 for (a) DJF PM_2.5_, JJA PM_2.5_, DJF PM_10_, and JJA PM_10_.

The percentage of days during DJF and JJA when simulated PM_2.5_ and PM_10_ concentrations exceed unhealthy levels for sensitive groups (> 35 μg/m^3^ for PM_2.5_ and 150 μg/m^3^ for PM_10_) is shown in Figures 6a–6d. During DJF 2015–2016, more than 90% of the simulated daily values of PM_2.5_ exceed moderate levels in the northern administrative districts of Saint Louis and Louga, with the administrative districts of Dakar, Thies, and Dourbel, which have large urban centers, showing more than 80% of the simulated days exceeded moderate air quality levels for PM_2.5_. There is a significant improvement during JJA, with only the administrative district of Saint Louis showing the simulated number of days for PM_2.5_ with unhealthy values exceeding 90%. Similar to PM_2.5_ exposure, PM_10_ concentrations exceeding 150 μg/m^3^ are found more than 90% of the winter season days in 2015–2016 in the northern administrative districts of Saint Louis and Louga (Figure 6c). In the southern administrative districts, moderate PM_10_ concentrations are found 40–60% of the winter seasons of 2015–2016. The spatial patterns for PM_10_ exposure is nearly the same as for PM_2.5_ during the summer season across Senegal with Saint Louis having the highest exposure (Figure 6d).

### 3.4 Total Number of Asthma, Bronchitis, and ARI Cases in Senegal During 2015 and 2016

**Figure 7:**
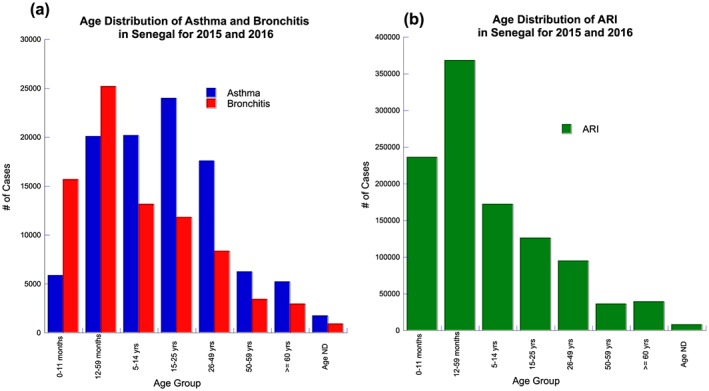
Age distribution for all cases in 2015 and 2016 for a asthma and bronchitis, and b ARI.

Because of the poor air quality in Senegal during the winter season, we wanted to examine the number of asthma, ARI, and bronchitis cases. The age distribution for asthma, bronchitis, and ARI reported cases during 2015 and 2016 is shown in Figures 7a and 7b. Asthma cases are found from ages in the ranges 12 months to 49 years of age with the largest number of cases in the 15‐ to 25‐year age group. Bronchitis and ARI cases have the highest values between 12 and 59 months and then decrease with increasing age. With respect to children cases (under 15), the age groups of 12–59 months account for 20% of the asthma cases, 31% of the bronchitis cases, and 34% of ARI cases. Specifically, for childhood asthma, 12.7%, 43.6%, 43.8% of the cases occurred for the age groups of 0–11 m, 12–59 m, and 5–14 years. In the case of childhood bronchitis, 29%, 46.6%, and 24.4% of the cases occurred for the age groups of 0–11 m, 12–59 m, and 5–14 years. In the case of childhood ARI, 30.4%, 47.4%, and 22.2% of the cases occurred for the age groups of 0–11 m, 12–59 m, and 5–14 years.

While bronchitis and ARI adult cases (> 14 years of age) are smaller than children, they are still substantial. For example, the highest number of asthma cases occurred for the age group of 15–25 years with more than 20,000 cases reported during 2015 and 2016. The number of adult ARI cases for ages greater than 15 years of age was significant with more than 100,000 cases during 2015 and 2016. Next, we discuss the temporal and spatial patterns of adult and children ARI, asthma, and bronchitis cases across Senegal for 2015 and 2016.

### 3.5 Temporal and Spatial Patterns of Adult Asthma, Bronchitis, and ARI During 2015–2016

**Figure 8:**
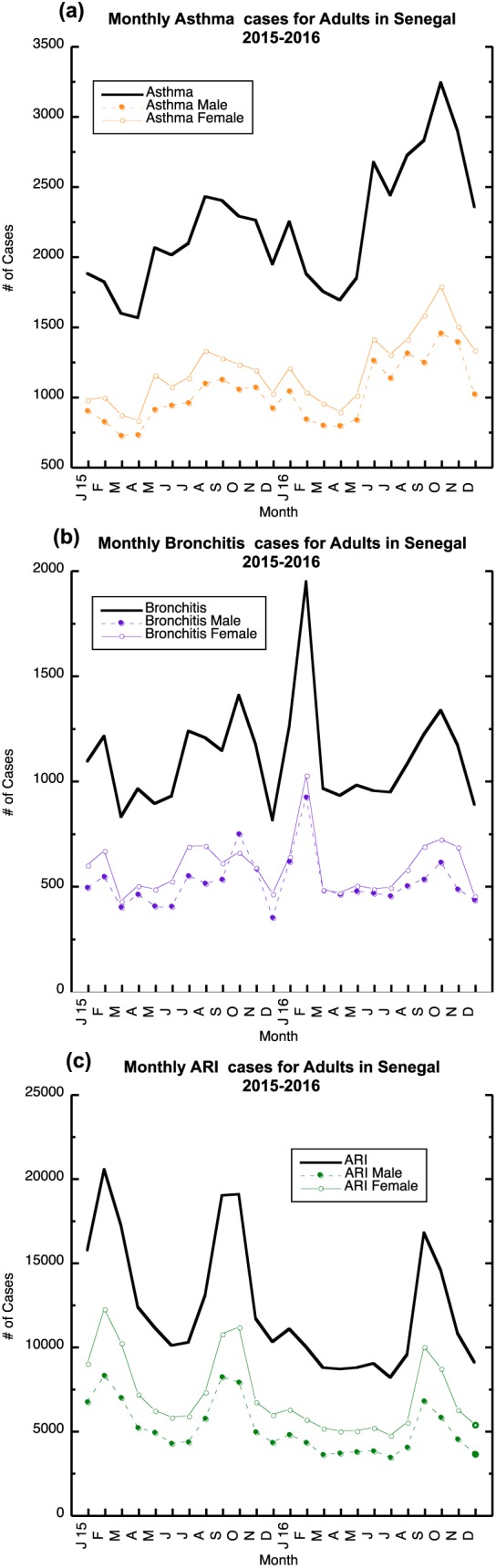
Monthly reported total, male and female adults (15–60) cases for 2015–2016: A asthma, b bronchitis, and c ARI.

Figures 8a–8c show the temporal distribution by gender for asthma, bronchitis, and ARI in Senegal for 2015 and 2016. Female adults in 2015 and 2016 had a higher prevalence of asthma, bronchitis, and ARI (Figures 8a–8c). In 2016, there is a sizable increase in the numbers of asthma cases starting in July through October for female and male adults; conversely, in 2015, there is a decline in cases after August during the same period (Figure 8a). Bronchitis cases tend to show more female cases with the largest number of cases occurring during February 2016. Additional peaks in bronchitis occur during the summer and fall of 2015 and the autumn of 2016 (Figure 8b). ARI also shows a larger number of female cases reported throughout 2015 and 2016, with peaks occurring in February, September, October of 2015, and September 2016 (Figure 8c). During 2015 and 2016 the percentage of female adult cases are 54% asthma, 53% bronchitis, and 58% for ARI. This higher percentage of female respiratory cases could have a significant household, child raising, and work‐related consequences for Senegal.

**Figure 9:**
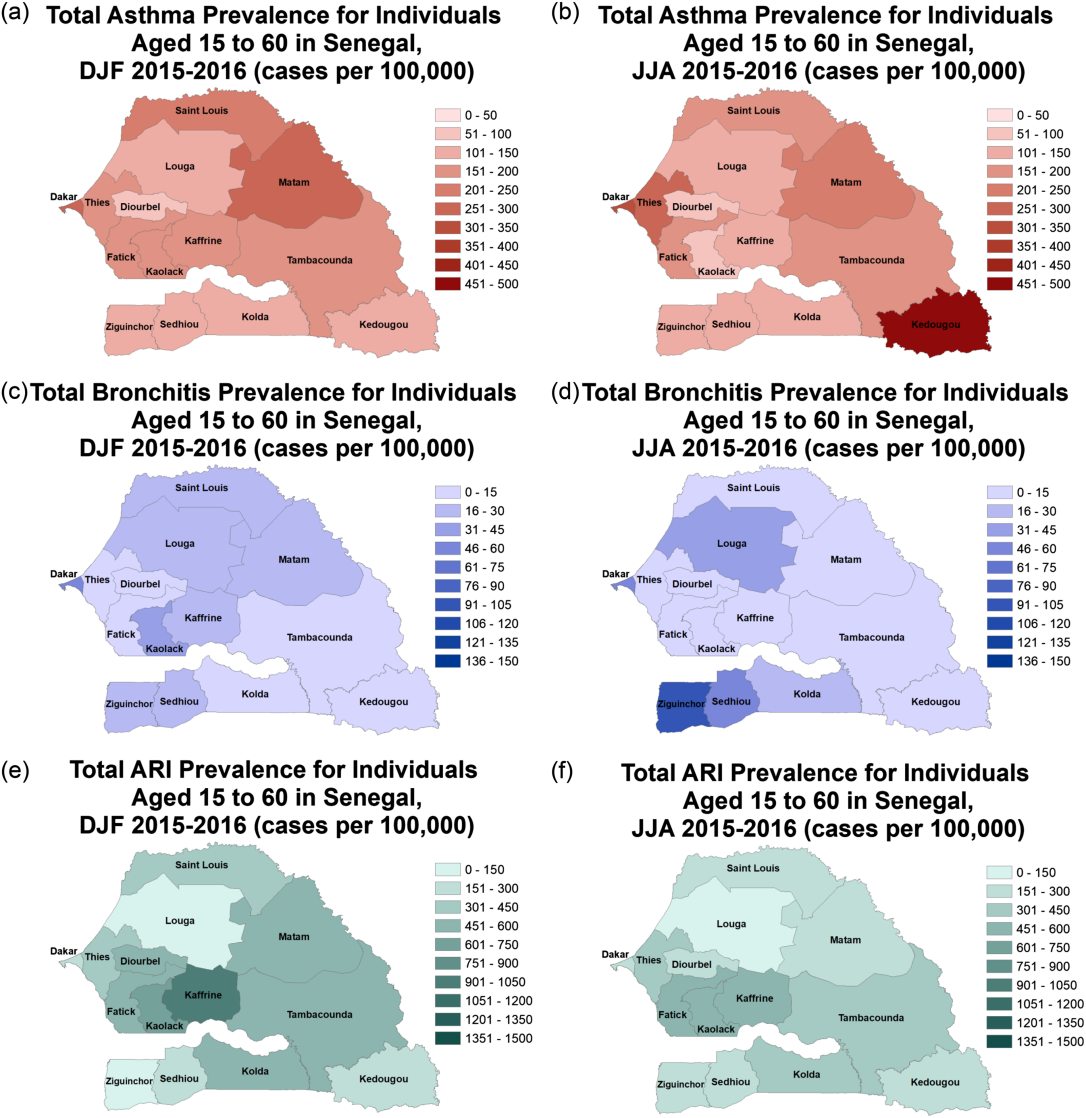
(a–f) DJF and JJA 2015 and 2016 computed adult disease prevalence across the 14 administrative districts for (a) DJF asthma, (b) JJA asthma, (c) DJF bronchitis, (d) JJA bronchitis, (e) DJF ARI, and (f) JJA ARI.

Figures 9a–9f show the DJF and JJA spatial distribution of asthma and bronchitis for adults of 15–60 years of age. During DJF the highest winter season prevalence in asthma was found in the administrative districts of Matam and Dakar (Figure 9a). During JJA, the highest asthma prevalence is found in the southeastern district of Kedougou followed by Dakar and Thies (Figure 9b). Additional sources of pollutants in capital city Dakar may be responsible for high asthma prevalence even when dust concentrations are reduced. The administrative districts with the highest bronchitis prevalence are Dakar, Louga, and Kaolack during DJF (Figure 9c). During the summer months, in addition to Dakar and Louga, the highest bronchitis prevalence is found in the southern administrative districts of Ziguinchor, Sedhiou, and Kolda (Figure 9d). Relative to asthma and bronchitis, higher prevalence values of ARI are found for adults in Senegal. During DJF the highest values are found in Kaffrine followed by Kaolack during 2015–2016 (Figure 9e). Higher prevalence values of ARI are also found in Fatick, Tambadounda, and Kolda during DJF. A reduction in JJA ARI prevalence is found in many administrative districts relative to DJF, especially Kaffrine (Figure 9f).

### 3.6 Temporal and Spatial Patterns of Children (<15 Years) Asthma, Bronchitis, and ARI During 2015–2016

**Figure 10:**
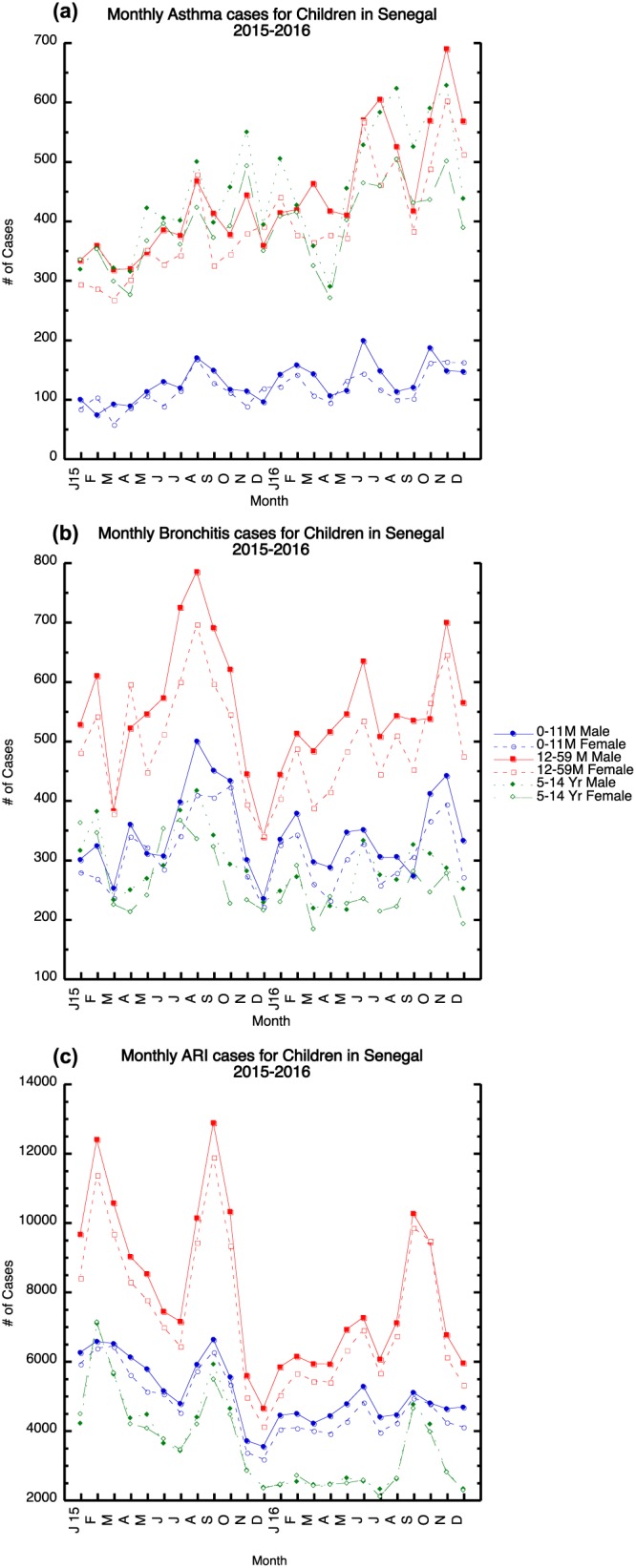
Monthly reported total, male and female children (0–11 months, 12–59 months, and 5–14 years) cases for 2015–2016: (a) asthma, (b) bronchitis, and (c) ARI. Monthly 2015–2016 Male and female children distribution of (a) asthma (b) bronchitis; (c) ARI.

Figure 10a shows the monthly distribution of asthma during 2015 and 2016 for children in the 0–11 m, 12–59 m and 5–14 year age ranges. For monthly asthma, 0–11 m male children have the smallest number of cases, while older children in the 12–59 m and 5–14‐year olds have a larger number of cases that are similar in size. In general, we find an increasing number of asthma cases from April through November in both years for the age groups of 12 months through 14 years of age (Figure 10a). In contrast to the adult population, more male children cases are reported during 2015 and 2016.

In 2015–2016, we also find the 12–59‐month male and female children bronchitis cases are higher than the other age groups with a winter and summer peak during 2015 and late spring and late autumn peak in 2016; the late autumn bronchitis peak in 2016 overlaps with the asthma peak. The numbers of ARI greatly exceed asthma and bronchitis cases (Figure 10c) during 2015 and 2016 with children in the 12‐ to 59‐month age range producing the largest number of cases. Several peaks are found in February 2015, September, October 2015, and November of 2016. A significant reduction in the number of children ARI cases relative to 2015 is found from January through August 2016, but this is especially true for in the age range of 5–14 (Figure 10c). The children ARI peaks occur at the same time as adults suggesting that infections could be pervasive across all age groups. Further, the ARI peaks in 2015 and 2016, for children older than 12 months, tend to occur during the period when increases in the number of bronchitis cases occur.

**Figure 11:**
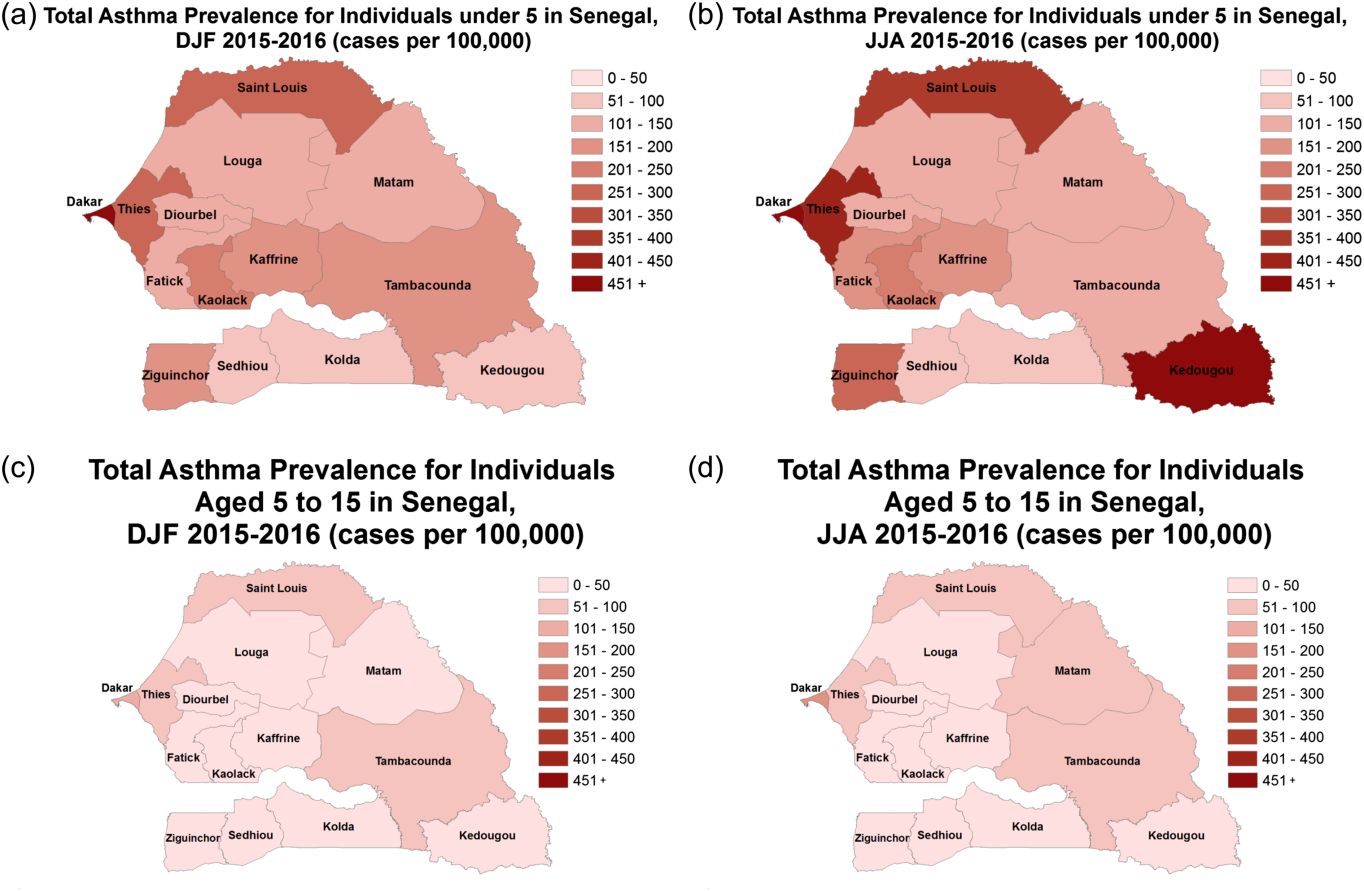
DJF and JJA 2015 and 2016 computed children disease prevalence across the 14 administrative districts for (a) DJF asthma under 5, (b) JJA asthma under 5, (c) DJF asthma 5–14, and (d) JJA asthma 5–14.

The spatial patterns of asthma for children under 5 years old and between the ages of 5 and 14 during DJF and JJA of 2015 and 2016 are shown in Figures 11a–11d. For children under 5 years during DJF the prevalence of asthma is highest in urbanized administrative districts of Dakar, Thies, and Saint Louis during the winter season (DJF, Figure 11a). During the summer season (JJA) the prevalence of asthma increases in these administrative districts and expands to include Kedougou and Ziguinchor (Figure 11b). During the winter and summer seasons, older children between 5 and 14 have a lower prevalence of asthma with the highest values found in the administrative districts of Dakar, Thies, Saint Louis, Tambacounda, and Matam (Figures 11c and 11d).

**Figure 12:**
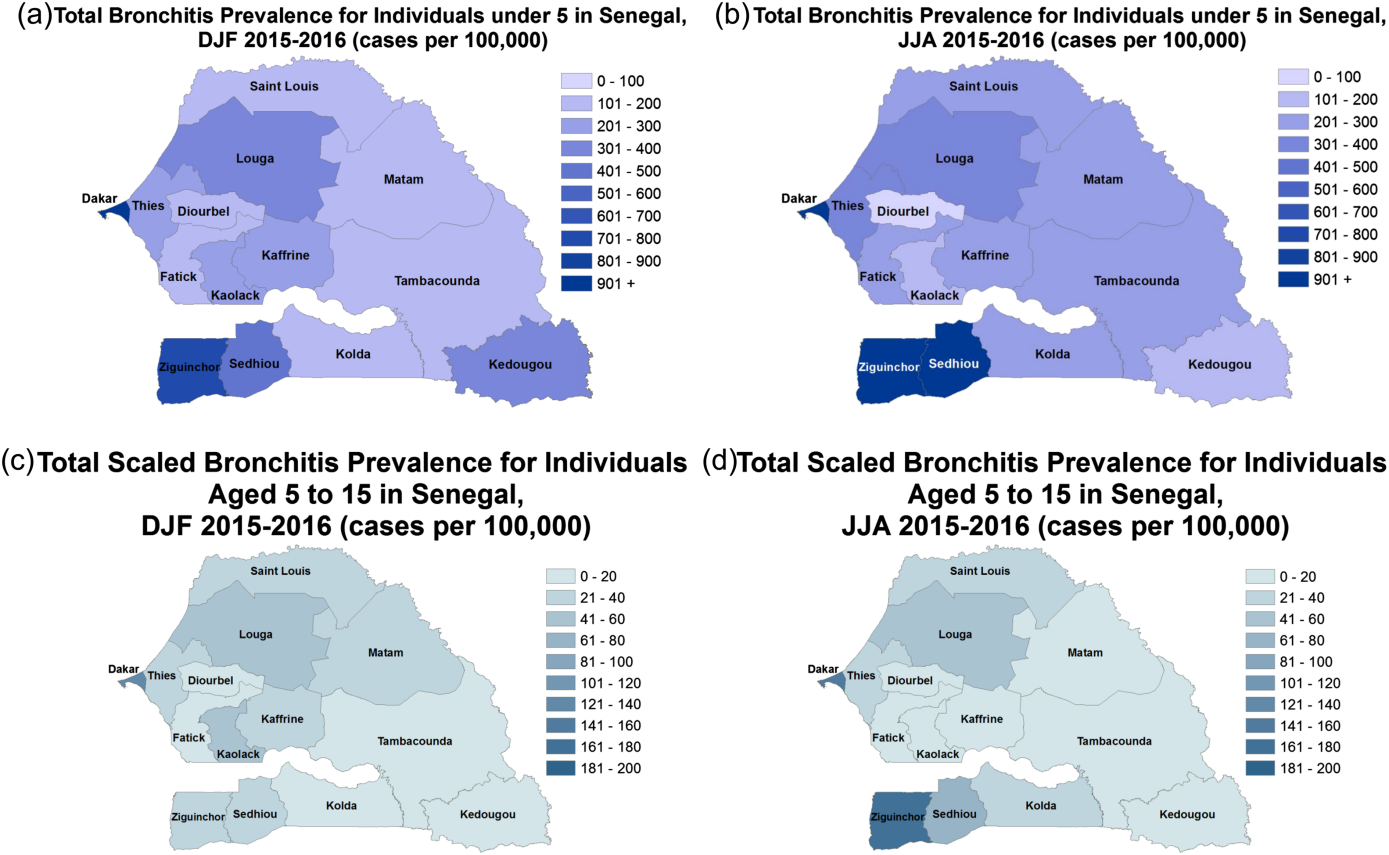
Same as Figure 11 but for bronchitis.

During DJF, the highest prevalence of bronchitis is found in the administrative districts of Dakar and to the south in Ziguinchor and Sedhiou for children under five (Figure 12a). During JJA there tends to be a higher prevalence of bronchitis across much of Senegal, except in the administrative districts of Diourbel, Kedougou, and Kaolack where decreases in prevalence are shown in Figure 12b. The prevalence of bronchitis is lower during both seasons for older children in the 5–15 age group. The highest values are found in the administrative district of Dakar during DJF and JJA and in Ziguinchor during JJA (Figures 12c and 12d).

**Figure 13:**
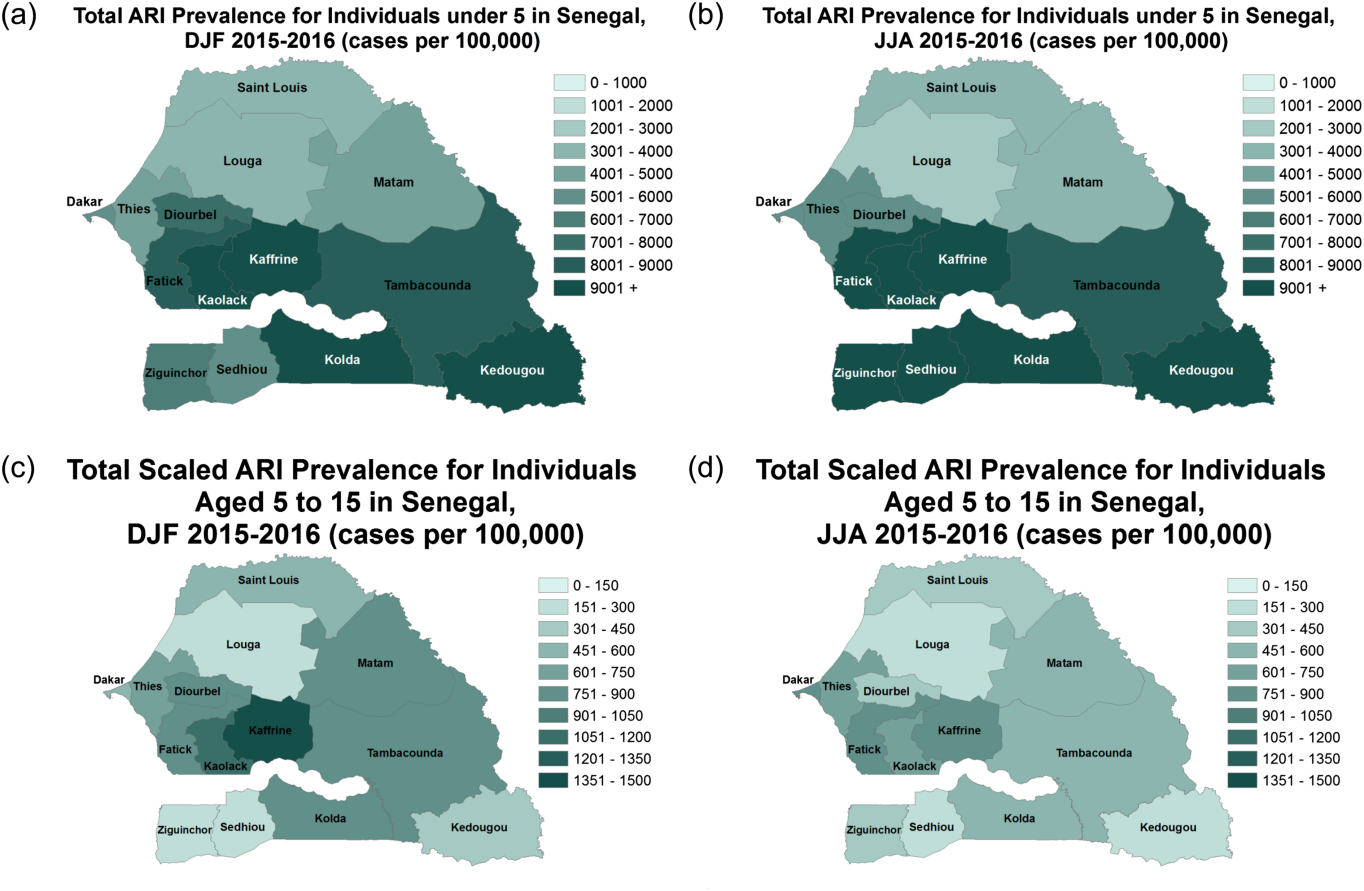
Same as Figure 11 but for ARI.

The prevalence of ARI for children under 5 years of age is alarmingly high over the central and southern administrative districts of Senegal during winter (DJF) and summer (JJA) seasons of 2015 and 2016 (Figures 13a and 13b). The administrative districts of Kaffrine, Kaolack, and Kedougou have more than 9,000 cases per 100,000 during the winter season (Figure 13a). During the summer season, nearly all of the southern administrative districts have very high ARI prevalence (Figure 13b). The prevalence of ARI for ages under 5 is smaller in the administrative district of Dakar relative to other areas, suggesting that ARI may not be driven by urban pollution. A lower prevalence of ARI occurs in the northern administrative districts during DJF and JJA. For older children of age of 5–14, there is a reduction in the prevalence of ARI similar to asthma and bronchitis. The highest prevalence of ARI is for the administrative district of Kaffrine during the winter season, and the eastern administrative districts of Matam and Tambacounda also have shown higher ARI prevalence during the winter season (Figure 13c). During the summer season, there is a reduction in the prevalence of ARI for ages 5–15 across Senegal. The highest values occur in the administrative districts of Kaffrine, Fatick, Dakar, and Thies (Figure 13d).

### 3.7 Observations of Tuberculosis in Senegal

- National

**Figure 14:**
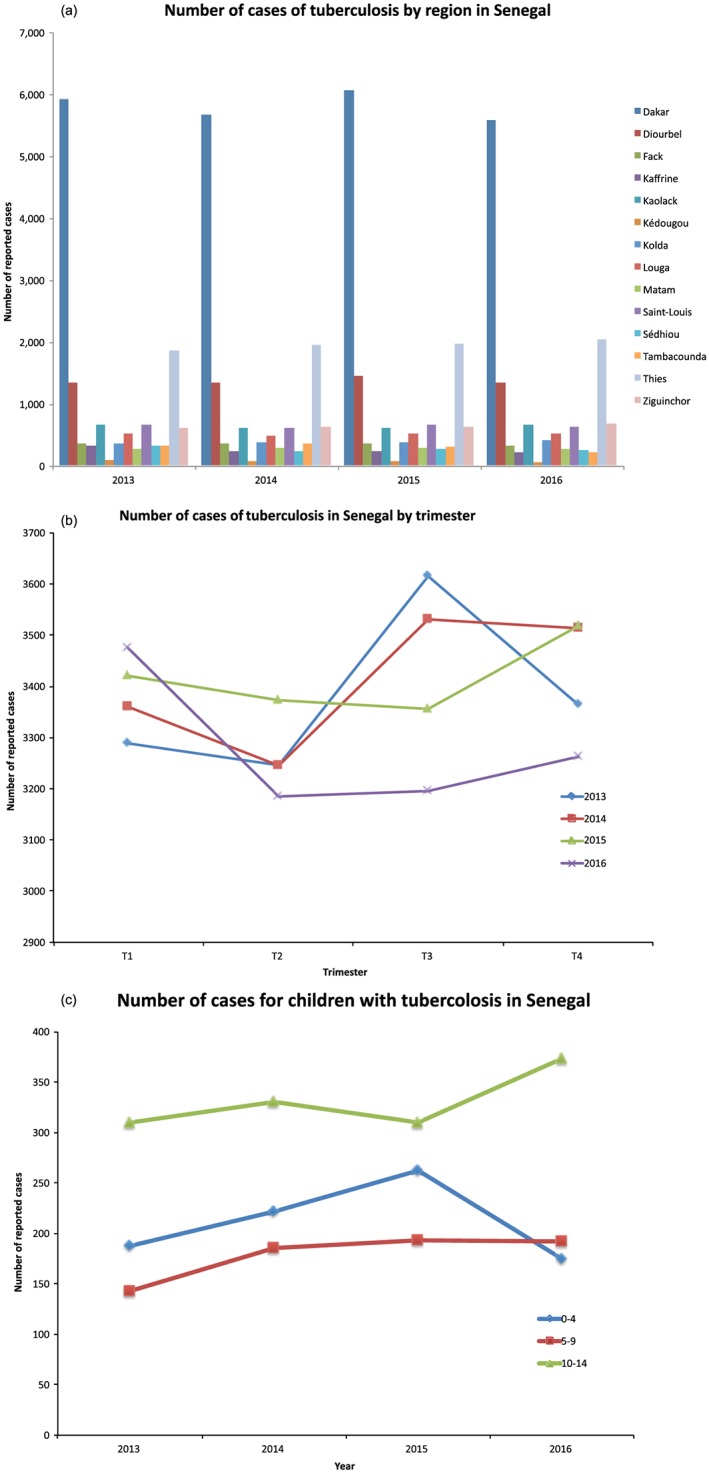
The 2013–2016 TB cases (a) by region, (b) by trimester, and (c) children by age.

The national program fighting against tuberculosis (PNT) is a response by Senegal to address the tuberculosis pandemic. The fight against tuberculosis appears as part of the defined priorities of the Ministry of Health and Social Action. The number of reported cases in the country, from 2013 to 2016, is 13513, 13098, 13667, and 13116. However, when it comes to the regions, they do not follow a uniform pattern with more than half of the reported cases in the administrative district of Dakar (Figure 14a). However, administrative districts with urban population are also found to have larger numbers of reported cases that includes Thies and Diourbel. The incidence rate of tuberculosis is around 170 to 200 new cases/100,000 inhabitants per year for tuberculosis in all forms and from 120 to 140 new cases/100,000 inhabitants per year for the smear‐positive pulmonary tuberculosis. This number has increased the most for male patients relative to female patients; the sex ratio is 2:1 for 2015. Data from 2001 to 2015 indicated that tuberculosis significantly affected the young to middle‐aged population (15–44 years); almost 80% of these reported cases are under the age of 45.

The distribution by trimester (January–March, April–June, July–September, and October–December) of these reported cases in Senegal shows that the number of cases is highest in the third trimester of 2013, the third and fourth trimesters of 2014, fourth trimester of 2015, and the first trimester of 2016 (Figure 14b). Children with tuberculosis represent 10% to 20% of all forms of cases, and the age group that is very vulnerable to these serious forms is that of 0–4 years (World Health Organization, 2012). In Senegal, TB in infants is underdiagnosed particularly in the regions with poverty. Despite the actions of the national program to fight against tuberculosis in Senegal (services are put into place to guide the charge against TB in children, screening algorithms, training services in treating children, testing with tuberculin to check the standard in communities, and giving free medications), there has been a stagnation in the rates of screenings of TB for those in the age range of 3 years of age (PNT‐Sénégal, 2013, 2014, 2015, 2016). The most commonly reported age group with tuberculosis is 10 to 14 years old (Figure 14c). This is probably related to a greater ability to collect sputum in children of this age.

b. Dakar.

**Figure 15:**
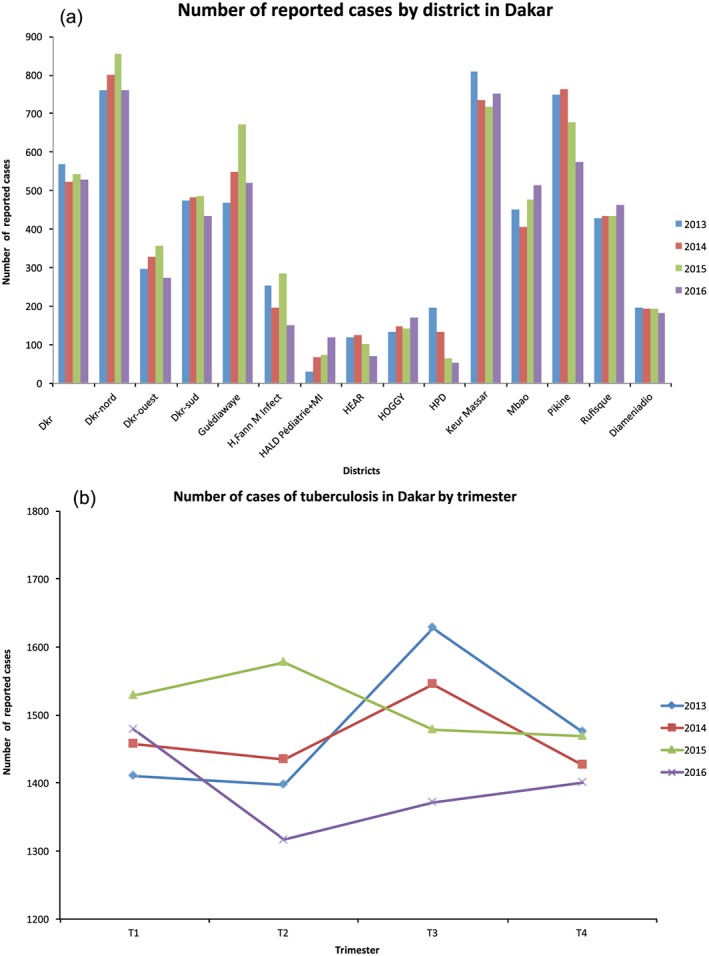
The 2013–2016 TB cases for Dakar (a) by district and (b) by trimester.

The administrative district of Dakar carries 22.4% of the total population in Senegal, but it is associated with more than 50% of reported tuberculosis cases. Within the administrative district of Dakar, the largest number of cases includes northern Dakar, Keur Massar, and Pikine (Figure 15a). The seasonality of TB follows a trend similar to the national trend. In general, the cases have a range of 1,300 to 1,600 cases per trimester during the 4‐year period. In trimester 1 (January–March) the number of cases is from 1,400 to 1,500 reported cases in 2013–2016. Trimester 2 (April–June) finds that the number of reported cases fall and peak around the same bracket of 1,300–1,600 cases. In trimester 3 (July–September) the number of reported cases is reversed from trimester 2 before coming to a similar trend, and in trimester 4 (October–December) the number of cases is similarly lower. The seasonality of tuberculosis cases fall between high and lower reported cases in the second and third trimesters. While in the first and fourth trimesters the number of reported cases is lower in all 4 years.

c. TB cases at the Pulmonology Clinic at Fann Hospital.

At Fann Hospital in the city of Dakar, patients with tuberculosis are usually treated as outpatients. Only those with complicated forms are hospitalized in the special department such as the Pulmonology Center at the Cheikh Anta Diop University‐Fann National Hospital in Dakar, Senegal. Approximately 418 cases of tuberculosis were found between 1 August 2015 and November of 2016. On most days, there were one to seven cases that occurred year round (not shown).

## 4 Discussion and Conclusion

Air quality, with an emphasis on PM, and its impact on respiratory health in Senegal during the period of 2013–2016, has been examined in this paper. Senegal and most of West Africa have very limited temporal and spatial surface measurements of PM. The majority of measurements in Senegal occur in the capital city of Dakar, and to address this issue, we have used the WRF model to estimate monthly PM_2.5_ and PM_10_ concentrations throughout the 14 administrative districts.

The PM_10_ and PM_2.5_ observations in Dakar, Senegal, from 2013 to 2016, show that there are many instances of unhealthy and even hazardous PM levels during the winter season. The PM levels are 7–8 times the maximum values observed in Barbados and can last for several days. Simulations of dust suggest that the northern parts of Senegal receive the highest PM_2.5_ and PM_10_ concentrations during the winter season, and that in some administrative districts, more than 90% of the days have values exceeded 150 μg/m^3^ for PM_10_ and 35 μg/m^3^ for PM_2.5_. Simulated PM_10_ and PM_2.5_ dust concentrations are considerably lower during the summer season, promoting good air quality over much of Senegal. From the vantage point of satellite‐based dust loading, the largest amounts are found during the summer season when surface PM measurements are at their lowest values.

Using limited health data during 2015 and 2016, we find (a) the highest prevalence of asthma, bronchitis, and ARI for children under 5 years of age; (b) Dakar carries the burden of asthma and bronchitis in the country, which suggests that there are multiple sources of pollution impacting human health; (c) young adults in the age group of 15–25 carry the highest adult burden of asthma; (d) the age group of 12–59 months has the highest burden of asthma, bronchitis, and ARI for children; (e) adult females carry a higher burden of ARI and bronchitis in Senegal; (f) ARI prevalence is very high in central and southern administrative districts of Senegal, exceeding 8,000 cases per hundred thousand in Kaffrine for children under 5 years of age; adult ARI prevalence is also high in Kaffrine; (g) seasonal changes in TB are not observed suggesting a limited impact from dust events.

The strongest linkages between air quality, asthma, and bronchitis are for urban areas and suggest that multiple factors such as traffic, industrial pollution, and household factors may exacerbate these diseases. However, the linkages between air quality are weaker in less populated administrative districts, for example, with higher a prevalence of adult and childhood asthma found in Dakar, Thies, Kedougou, and Saint Louis during JJA when PM concentrations are reduced. Limited studies of asthma in six West African countries have been undertaken by To et al. (2012) using surveys and have found the highest prevalence in Mauritania with Senegal having the second highest prevalence of wheezing symptoms. Other environmental factors, such as higher temperature or relative humidity, may influence asthma outside the dusty season, as is the case in Barbados (Prospero et al., 2008). Given the observed high PM_10_ and PM_2.5_ concentrations in Dakar and the suggestion from model simulations that values are even higher over the northern districts, it is imperative that PM observations are increased in Senegal especially in these regions. Additional sources of PM (biomass burning, urban pollution, and indoor cooking pollution) that can impact human must also be taken into accounts to determine the impacts on human health. A country‐wide PM ground‐based network in Senegal can help to improved forecasted and simulated PM and improve the relationship between satellite‐based AOD and surface PM_2.5_ and PM_10_ concentrations. Given that a significant fraction of infant mortality maybe linked to PM_2.5_, improved forecasting of PM_2.5_ may help in reducing PM‐related infant mortality through increased awareness and warning to limit outdoor activities when hazardous conditions arise.

## CONFLICT OF INTEREST

The authors declare no conflicts of interest relevant to this study.
